# *Staphylococcus aureus* Infection Reduces Nutrition Uptake and Nucleotide Biosynthesis in a Human Airway Epithelial Cell Line

**DOI:** 10.3390/metabo6040041

**Published:** 2016-11-09

**Authors:** Philipp Gierok, Manuela Harms, Karen Methling, Falko Hochgräfe, Michael Lalk

**Affiliations:** 1 Institute of Biochemistry, University of Greifswald, 17487 Greifswald, Germany; gierokp47@uni-greifswald.de (P.G.); methling@uni-greifswald.de (K.M.); 2 Competence Center Functional Genomics, Junior Research Group Pathoproteomics, University of Greifswald, 17487 Greifswald, Germany; manuela.harms@uni-greifswald.de (M.H.); falko.hochgraefe@uni-greifswald.de (F.H.)

**Keywords:** host cell metabolism, infection, metabolomics, *Staphylococcus aureus*

## Abstract

The Gram positive opportunistic human pathogen *Staphylococcus aureus* induces a variety of diseases including pneumonia. *S. aureus* is the second most isolated pathogen in cystic fibrosis patients and accounts for a large proportion of nosocomial pneumonia. Inside the lung, the human airway epithelium is the first line in defence with regard to microbial recognition and clearance as well as regulation of the immune response. The metabolic host response is, however, yet unknown. To address the question of whether the infection alters the metabolome and metabolic activity of airway epithelial cells, we used a metabolomics approach. The nutrition uptake by the human airway epithelial cell line A549 was monitored over time by proton magnetic resonance spectroscopy (^1^H-NMR) and the intracellular metabolic fingerprints were investigated by gas chromatography and high performance liquid chromatography (GC-MS) and (HPLC-MS). To test the metabolic activity of the host cells, glutamine analogues and labelled precursors were applied after the infection. We found that A549 cells restrict uptake of essential nutrients from the medium after *S. aureus* infection. Moreover, the infection led to a shutdown of the purine and pyrimidine synthesis in the A549 host cell, whereas other metabolic routes such as the hexosamine biosynthesis pathway remained active. In summary, our data show that the infection with *S. aureus* negatively affects growth, alters the metabolic composition and specifically impacts the de novo nucleotide biosynthesis in this human airway epithelial cell model.

## 1. Introduction

The Gram positive bacterium *Staphylococcus aureus* is not only a permanent commensal of about 20% of the world population but also an opportunistic pathogen [1,2]. Infections can result in diverse clinical manifestations such as soft local tissue infections, endocarditis, sepsis and also pneumonia [2,3]. *S. aureus* has been described earlier as an extracellular pathogen that exhibits its pathogenicity with the secretion of virulence factors [4]. Within the last two decades, however, *S. aureus* has also been recognized as an invasive pathogen with an intracellular lifestyle [5,6]. It was shown by proteomic and transcriptomic studies that intracellular *S. aureus* undergoes changes in expression of metabolic genes, nutrient transporters and virulence factors to adapt to the intracellular environment [7,8]. 

To prevent colonization in the human lung, the respiratory epithelium maintains an effective antimicrobial environment. This is accomplished by various antimicrobial strategies such as forming a physical barrier, mucociliary clearance, and production of antimicrobial peptides, surfactant proteins, complement, chemokines, and cytokines [9,10,11]. Many of these defence mechanisms are activated by staphylococcal virulence factors [12] but so far only little is known about the consequences on the host cell metabolism. Recently, we described the effect of staphylococcal alpha toxin (Hla) on glycolysis and glutaminolysis of human airway epithelial cells [13]. Although this study shows that the host metabolism is affected by the action of single virulence factors, the complex process of infection might influence the host cell metabolism very differently. During the invasion process *S. aureus* adherence proteins such as fibronectin binding proteins bind to host cell structures such as α_5_β_1_ integrin via fibronectin and induce a zipper-type uptake [14]. The uptake activates the rearrangement of the cytoskeleton [15] and several regulators that are also involved in metabolism such as the PI3K-Akt pathway [16,17,18]. Moreover, cellular processes that are directly coupled to the host metabolism such as autophagy [19] and apoptosis [20] are affected by *S. aureus.* In between this complex interplay of cellular processes and signalling events metabolites serve as signal molecules, precursors for antimicrobial effector molecules and also fuel primary anabolic and catabolic pathways. From the view of the intracellular pathogen the host cell metabolome represents a source of nutrients [21]. Interestingly, only adapted bacteria are able to grow in this environment [22]. Therefore, alterations in the host cell metabolite composition also affect the intracellular pathogen.

In this work the host cell metabolome of A549 human airway epithelial cells was examined and the effect of the infection with *S. aureus* was elucidated on the intracellular and extracellular level. We observed in infected A549 cells a strongly reduced uptake of nutrients, especially of essential amino acids. Moreover the analysis of the intracellular metabolic profiles in a time dependent manner showed dynamic changes in the content of free amino acids and certain nucleotides. Furthermore, we elucidated that the de novo synthesis of purine and pyrimidine nucleotides is shut down after infection by using metabolic inhibitors and a metabolic labelling approach. 

## 2. Results

### 2.1. A549 Cells Enter Growth Arrest after Exposure to S. aureus

**Figure 1:**
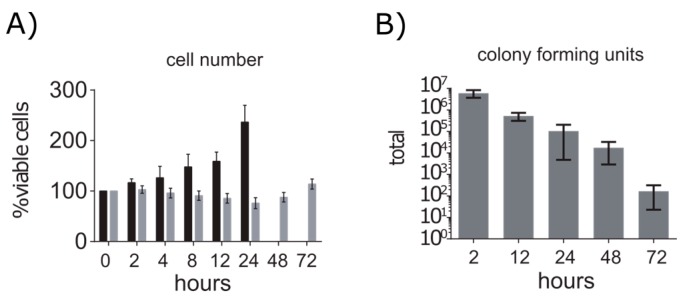
Cell numbers of A549 cells and *S. aureus* during infection. (**A**) Growth of infected (grey) and control A549 cells (black) is displayed in percentages with 100% being the initial cell number. Data are presented as mean with standard deviation (*n* = 5). (**B**) Total amount of colony forming units after indicated time points of infection. Data are presented as mean with standard deviation (*n* ≥ 6).

After the infection, A549 cells were incubated for 72 h and the cell number and the amount of intracellular *S. aureus* cells was monitored. We replaced the medium every 24 h to prevent nutrient limitation and to reduce the amount of dead cells since about 25% of the population died within the first 24 h after infection. Between 24 h and 48 h the cell number remained stable, whereas between 48 h and 72 h we observed an increase of 38% (Figure 1A). Simultaneously, we observed a constant decline in the amount of intracellular bacteria over 72 h (Figure 1B). The cell number of control cells doubled within 24 h after which maximum confluence were reached. These data indicate that the exposure of A549 cells to *S. aureus* led to cell death in a proportion of cells and to a temporary growth arrest. Since most dying host cells were probably infected, this would also explain the strong decline of the *S. aureus* cell number within the cell culture. 

### 2.2. Extracellular Metabolic Profiles of A549 Cells after Infection with S. aureus

**Figure 2:**
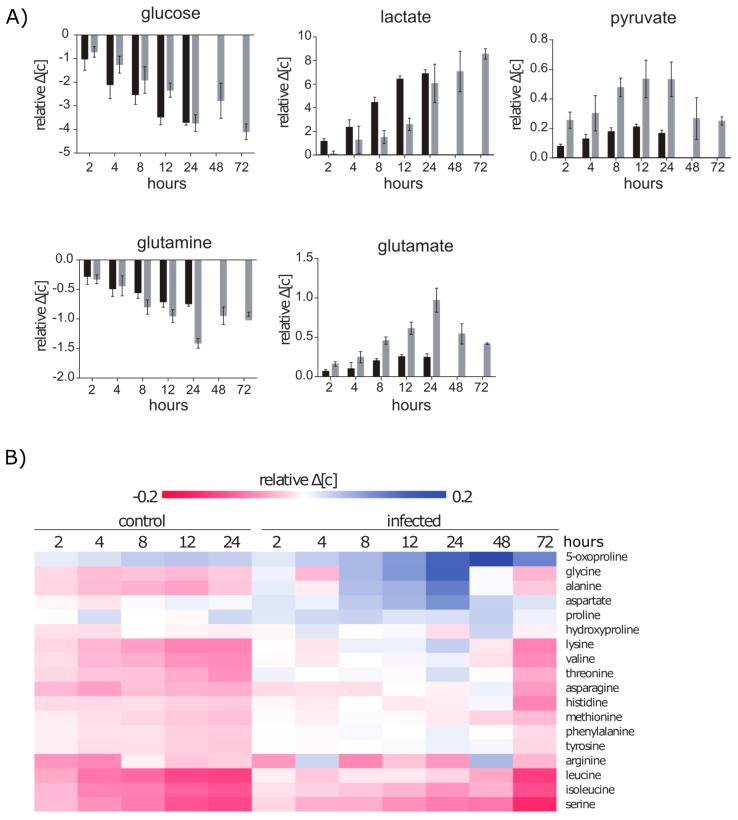
Extracellular metabolic profile during infection. (**A**) Extracellular concentrations changes of metabolites involved in glycolysis and glutaminolysis of control cells (black bars) and infected cells (grey) normalized on the proliferation rate ([c_tx_-c_t0_])/(cell number_tx_/cell number_t0_). Data are presented as mean with standard deviation (*n* ≥ 6). (**B**) Extracellular concentrations changes of other amino acids were normalized on the proliferation rate ([c_tx_-c_t0_])/(cell number_tx_/cell number_t0_). Data are presented as mean (*n* ≥ 6) in a colour coded chart. Red fields indicate an increase in the extracellular concentration and blue fields indicate a decrease of the metabolite concentration compared to the initial concentration.

By using ^1^H-NMR-spectroscopy we analysed the extracellular concentration of nutrients such as amino acids and glucose in the medium of control and infected cells (Figure 2). Thereby, we observed a reduced uptake of nutrients essential for growth and an infection related secretion of specific metabolites.

#### 2.2.1. Glucose and Glutamine Are Consumed by A549 under Control and Infection Conditions

Similar to other immortalized or cancer cell lines, A549 cells are expected to consume high amounts of glucose and exhibit a high glycolytic rate under aerobic conditions, which is known as the “Warburg effect” [23,24]. Furthermore, cancer cell metabolism requires an enhanced glutamine uptake to fuel anabolic pathways via glutaminolysis [25,26]. Indeed, we observed that 78% of the provided glucose and 87% of the provided glutamine were consumed by A549 cells under non-infection conditions within 24 h (Supplementary Material Table S1). In accordance to the high rates of glycolysis, we detected extracellular lactate in appropriate concentrations up to 18.14 mmol/L. This corresponds to approximately 1.85 molecules of lactate per metabolized glucose molecule. Additionally, extracellular pyruvate and glutamate showed increased concentrations of 0.39 mmol/L and 0.58 mmol/L over time respectively, indicating a secretion of these metabolites. The secreted amount of glutamate corresponds to 33% of the consumed glutamine. Both the pyruvate and the glutamate secretion suggest an intracellular overflow of intermediates of central metabolic carbon pathways. 

After infection with *S. aureus*, the A549 host cells maintained the glycolytic metabolism but only produced 1.62 molecules lactate per metabolized molecule of glucose. This indicates a slightly different intracellular flux of glucose under infection conditions. We compared the concentration differences between the medium and the sampling time points with regard to the factor with which cells had multiplied (Figure 2A). We found that infected cells use less glucose and produced less lactate within the first 12 h after infection. After that, the consumption of glucose and production of lactate was similar again to non-infected cells even if the growth was strongly reduced. Interestingly, the glutamine uptake was increased in infected cells significantly after 8 h and was elevated even more till 24 h. Moreover, between 2 h and 24 h after infection, pyruvate and glutamate secretion was strongly increased. However, after 48 h and 72 h glutamine uptake as well as glutamate and pyruvate secretion were reduced by approximately 30% and 50%, respectively. This indicates that the elevated secretion of glutamate and pyruvate is infection related and might represent an intracellular excess of these metabolic precursors.

#### 2.2.2. Amino Acid Uptake Is Reduced in the Infected A549 Cell Culture but the Secretion of Amino Acids Is Enhanced

Aside from glutamine and glutamate, we were able to quantify 15 more amino acids in the extracellular space. For most of these amino acids, a clear uptake was detected under control conditions in particular for the branched-chain amino acids and methionine (Figure 2B). After infection, however, the amino acid uptake was strongly reduced. Especially for threonine, phenylalanine, lysine and valine no uptake was detected within 24 h. Moreover, we found that concentrations of glycine, aspartate and alanine were increased in the extracellular space. This indicates that the production of amino acids is still active after the infection. However, the incorporation or usage of amino acids was presumably reduced and thus led to an intracellular overflow and consequently to the secretion. After 48 h the secretion was strongly reduced and between 48 h and 72 h the amino acid uptake was similar compared to control cells. Since the uptake of essential amino acids is essential for growing cells, we used cytochalsin D as an inhibitor of actin polymerization to inhibit cell growth in A549 cells and monitored the amino acid consumption. Surprisingly, growth inhibited cells did not show lower uptake rates compared to control cells (Figure S1A). This indicates that the reduced amino acid consumption under infection conditions is rather caused by the infection than a secondary effect of the growth arrest.

### 2.3. Amino Acids, Amino Acid Derivatives and Nucleotides Show Major Differences after Infection

**Figure 3:**
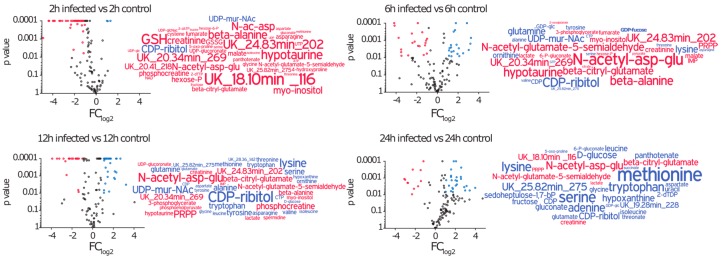
Time dependent metabolic profiles of A549 cells after exposure to *S. aureus*. Volcano plots display the log_2_ transformed fold change of intracellular metabolite amounts (normalized on 10^7^ cells) of infected versus control cells and the corresponding p-value (*n* = 3–9 out of at least three independent experiments). Metabolites with a *p*-value < 0.01 are coloured blue when FC_log2_ > 1 and red when FC_log2_ < −1. Significantly changed metabolites are displayed in word clouds in the corresponding colours and relative font size.

In this approach, we analysed in total 105 metabolites by using GC-MS and HPLC-MS (Supplementary Material Table S2). We compared the intracellular metabolic profile of infected cells at 2 h, 6 h, 12 h, and 24 h after infection with the corresponding control and observed significant differences over time (Figure 3). Two hours after the infection we found 45 metabolites to be altered with 43 being decreased and only two increased. The latter ones are CDP-ribitol and UDP-MurNAc, which are most likely of bacterial origin. Over time, however, the metabolic profile of infected cells got more diverse. After 6 h and 12 h we detected increased amounts for 18 and 24 metabolites respectively, whereas on the other hand some metabolites such as amino acid derivatives, *myo*-inositol and phosphocreatine remained at low concentrations. Twenty-four hours after infection, the majority of metabolites that were significantly different compared to control, were elevated in concentration.

#### 2.3.1. Intracellular Amino Acids Depleted in Growing Cells, but Accumulated in Infected Cells

**Figure 4:**
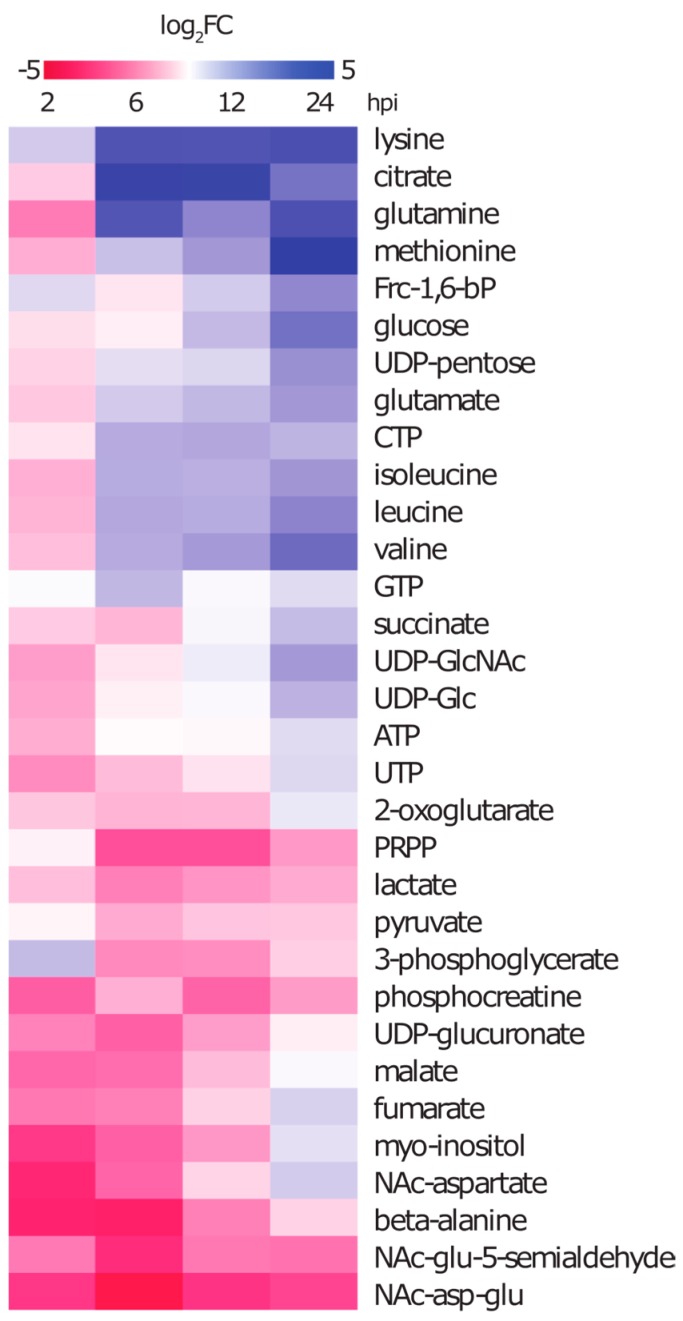
Fold change development of selected metabolites. Time dependent FC_log2_ (infected vs. control) of selected intracellular amino acids, metabolites of primary carbon metabolism, nucleotides and other identified metabolites are presented in a colour coded chart. Red indicates a lower amount and blue indicates a higher amount of the metabolite in infected cells compared to control cells.

Amino acids are important building blocks in growing cells and in accordance with that their extracellular concentration declined due to the consumption by the cells. Consequently, most amino acids show also an intracellular decrease in concentration during growth under control conditions. This is especially pronounced for methionine, which was neither detectable in the intracellular nor extracellular space of control cells after 24 h. However, under infection conditions, the intracellular amino acid profile showed a contrary development. Although, we found nine amino acids in significantly lower amounts 2 h after infection, most intracellular amino acid concentrations were found to increase significantly over time (Figure 4). Since this was not only true for non-essential but also for essential amino acids it is unlikely to be the result of enhanced amino acid production. Furthermore, the uptake of essential amino acids was clearly compromised by the infection. This led to the assumption that the rising amounts of intracellular amino acids may be accomplished by two factors: either by bacterial degradation or by host cell protein turnover due to proteasomal activity and cellular recycling processes such as autophagy.

#### 2.3.2. Metabolites of Central Carbon Metabolism Show Only Minor Changes under Infection Conditions

After 24 h, the intracellular glucose concentration in control cells was almost depleted in accordance with the significantly lowered extracellular amount of glucose. However, the intracellular glucose content in infected cells was six-fold higher at this time point compared to the control (Figure 4), which also corresponds to the higher extracellular glucose concentration. On the one hand, beside glucose other metabolites that are upstream in catabolic pathways such as fructose, hexose-P, gluconate and 6-P-gluconate were found in higher intracellular concentrations. On the other hand, some downstream glycolysis intermediates like 3-phosphoglycerate and phosphoenolpyruvate were found in decreased concentrations after 6 h compared to the control cells. Pyruvate and lactate showed also significantly lower concentrations, which corresponds to their secretion into the medium. Several metabolites of the tricarboxylic acid cycle showed a decreasing concentration during the first 12 h of infection compared to the control. Within the next 12 h, either the metabolite concentrations remained constant in infected cells and dropped in control cells (2-oxo-glutarate and malate), or rose in infected cells (succinate, fumarate, citrate and isocitrate).

#### 2.3.3. During Infection the Concentration of Secondary Metabolites Declines

Derivatives of amino acids like NAc-aspartate, NAc-aspartyl-glutamate, beta-citryl-glutamate and NAc-glutamate-5-semialdehyde were found to drop strongly in concentration 2 h after infection and only NAc-aspartate recovered to control amounts after 12 h (Figure 4). This indicates that the production of these compounds after infection is reduced. *Myo*-inositol, which is a very abundant metabolite in control cells was found to strongly decrease during the first 12 h of infection but recovered to control amounts before 24 h. Another metabolite that strongly declined under infection conditions was the energy storage compound phosphocreatine.

#### 2.3.4. The Nucleotide Profile Showed Elevated CTP and GTP Levels in Infected A549 Cells

We were able to detect the nucleotides involved in RNA and DNA synthesis together with intermediates of the purine and pyrimidine biosynthesis pathways. The nucleoside triphosphates required for RNA synthesis (ATP, UTP, GTP and CTP) were found to be the most abundant metabolites of these pathways. The amounts of ATP together with UTP and some of its sugar derivatives like UDP-glucose (UDP-Glc), UDP-*N*-acetyl-glucosamine (GlcNAc) and UDP-glucuronate (UDP-GlcA) were significantly decreased between 2 h and 6 h after infection. GTP and GDP increased 6 h after the infection together with CTP and CDP. Whereas, GTP dropped to control conditions again CTP, CDP and GDP remained elevated until 24 h (Figure 4). It is noteworthy that phosphoribosyl-pyrophosphate (PRPP), a key intermediate of purine and pyrimidine synthesis derived from the pentosephosphate-pathway (PP-pathway), was found in approximately seven-fold lower amounts 6 h after the infection and remained at that low level. 

### 2.4. Metabolic Profiles of Infected A549 Cells in the Presence of Glutamine Analogues Indicate a Different Enzyme Activity after Infection

In our metabolomics approach we found that CTP and GTP were elevated at time points during infection whereas ATP and UTP were decreased or rather unchanged. If increased CTP and GTP levels play a role during infection is so far unknown. In this approach, we tried to inhibit the synthesis of CTP and GTP by applying the glutamine analogues diazo-5-oxo-l-norleucine (DON) or azaserine to the infected cells. These compounds are known to inhibit glutamine transaminase reactions and glutamine is an important precursor and nitrogen donor in the de novo synthesis of purines and pyrimidines. 

#### 2.4.1. Inhibition of Purine and Pyrimidine Synthesis by DON and Azaserine

**Figure 5:**
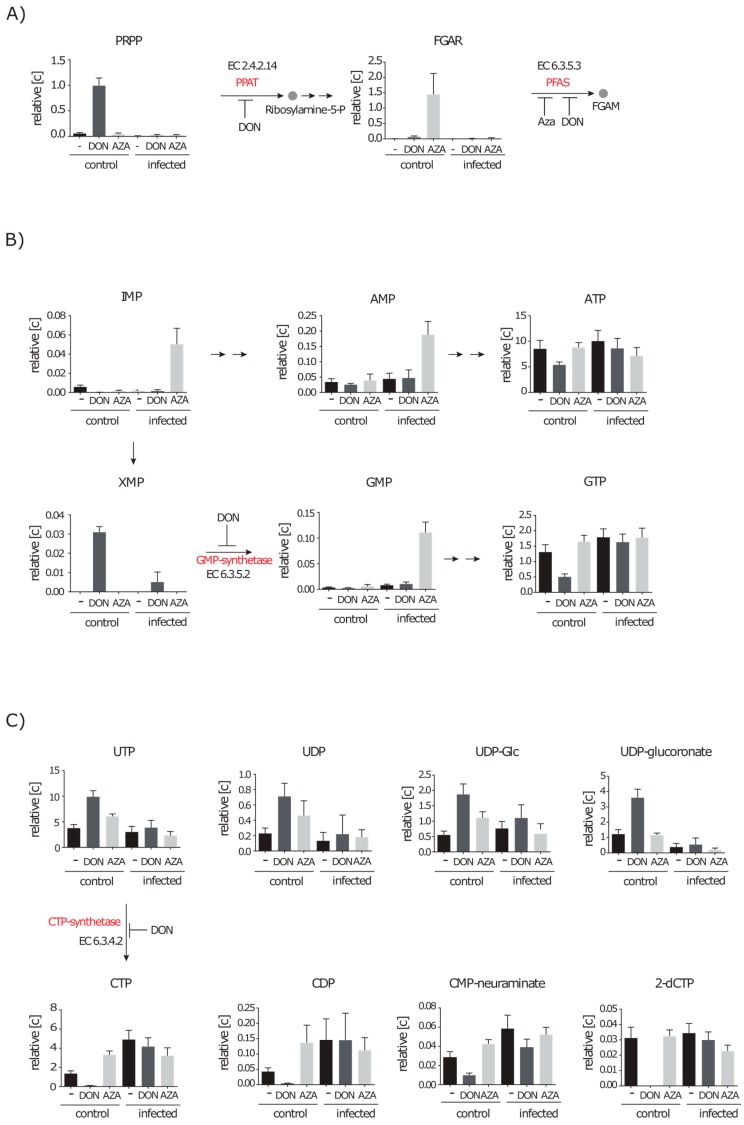
Inhibition of nucleotide synthesis with glutamine analogues. Control cells and infected cells were treated with either DON or azaserine (AZA) for 12 h. Identified educts and products of glutamine dependent reactions involved in purine and pyrimidine synthesis are displayed in relative concentrations as mean with standard deviation normalized on 10^7^ cells (*n* ≥ 7 out of at least four independent experiments). (**A**) Reactions of phosphoribosylpyrophosphate amidotransferase (PPAT) (EC 2.4.2.14) and phosphoribosylformylglycinamidine synthase (PFAS) (EC 6.3.5.3); (**B**) GMP-synthetase reaction (EC 6.3.5.2); and (**C**) CTP synthesis by CTP-synthetase (EC 6.3.4.2) and derivates of CTP and of the precursor UTP.

Since both inhibitors are reported to vary in their target enzymes and efficiency [27,28], we performed the infection experiment and added DON or azaserine in growth inhibitory concentrations afterwards to separate cultures. Due to the inhibition we expected educts of glutamine transferase reactions in increased concentrations. Indeed, the amount of PRPP, the educt of the glutamine transaminase reaction by the phosphoribosylpyrophosphate amidotransferase (PPAT), was increased 17 fold in uninfected A549 cells in the presence of DON (Figure 5A). The same is true for 5′-phosphoribosyl-*N*-formylglycinamide (FGAR) the educt of the transaminase reaction by the phosphoribosylformylglycinamidine synthase (PFAS) when cells were treated with DON (14 fold) or azaserine (300 fold) (Figure 5A). The accumulation of FGAR in DON treated cells was probably lower due to the additional upstream inhibition of the PPAT. 

Whereas PRPP was not increased in infected cells treated with the inhibitor, the FGAR level was also elevated eight fold in infected cells treated with azaserine. However, it was still only slightly above the detection limit. The next glutamine dependent reaction in purine synthesis is the conversion of XMP to GMP by the GMP-synthetase. XMP was solely detected in DON treated cells indicating an inhibition of this enzyme in the presence of DON but again not azaserine (Figure 5B). Despite the described potential inhibition sites in the purine synthesis under control conditions, ATP and GTP were still present in the cell but in 1.6- and 2.6-fold decreased amounts. Noteworthy, GMP, IMP and AMP amounts increased by 13, 30 and four fold, respectively, in infected cell cultures treated with azaserine. This indicates that azaserine only affects glutamine dependent reactions, but also might exhibit secondary effects that appear only under infection conditions (Figure 5B).

A glutamine using step in the synthesis of pyrimidines is the ligase reaction performed by the CTP-synthetase. A549 cells treated with DON showed strongly decreased amounts (12-fold lower) of the nucleotide CTP, which is an abundant metabolite under control and infection conditions (Figure 5C). Consequently, we found that CTP dependent metabolites also decreased in concentration. We detected CDP, the DNA precursor 2-deoxy-CTP and a proteoglycan precursor CMP-neuraminate in 13-fold, 18-fold and nine-fold lower amounts, respectively. Additionally, DON treated cells showed two- to three-fold higher amounts of UTP which is the direct precursor of CTP. Similar effects were observed for UTP derived metabolites such as UDP, UDP-Glc, UDP-GlcA and UDP-pentose. Azaserine on the other hand showed no inhibitory effect on CTP synthesis since a two- to three-fold increase in CTP and CDP concentration under control conditions was measured in treated cells. Interestingly, infected cells treated with either DON or azaserine showed no decline in CTP concentration but an increase up to 3.6 fold. 

These data show that: (i) azaserine has less target enzymes than DON in A549 cells at the chosen concentration; and (ii) infected cells are either less susceptible to inhibitor treatment, or the reactions that are supposed to be inhibited are not active under infection conditions. 

#### 2.4.2. Inhibition of the Hexosamine Pathway by DON under Control and Infection Conditions

**Figure 6:**
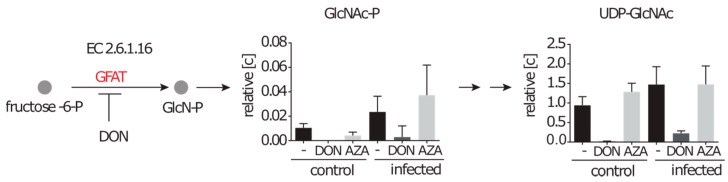
The hexosamine-pathway can be inhibited in infected cells. Control cells and infected cells were treated with either DON or azaserine (AZA) for 12 h. Successor metabolites of the glucosamine-fructose-6-phosphate aminotransferase reaction (GFAT) (EC 2.6.1.16) are displayed in relative concentrations as mean with standard deviation normalized on 10^7^ cells (*n* ≥ 7 out of at least four independent experiments).

Another anabolic pathway that relies on glutamine as a nitrogen donor is the hexosamine biosynthesis pathway (HBP). *N*-glucosamine-P (GlcN-P), a precursor of nucleotide sugars, is generated by a glucosamine-fructose-6-phosphate aminotransferase (GFAT) from fructose-6-P and glutamine. It is further acetylated to GlcNAc-P, which we were able to detect in low amounts under control conditions without inhibitor. After DON treatment, however, the intracellular GlcNAc-P was abolished (Figure 6). GlcNAc-P together with UTP can be converted to UDP-GlcNAc, a key metabolite in proteoglycan synthesis and protein modification. A 43-fold decrease in the UDP-GlcNAc amount was observed when DON was applied, thereby strongly indicating that the GFAT is inhibited by DON. In contrast to the previous described inhibited reactions of the purine and pyrimidine synthesis, we observed also strongly decreased amounts of GlcNAc-P and UDP-GlcNAc concentrations under infection conditions (eight fold and seven fold, respectively). 

#### 2.4.3. DON Affects Glutaminolysis and Azaserine the Central Carbon Metabolism in Infected Cells

**Figure 7:**
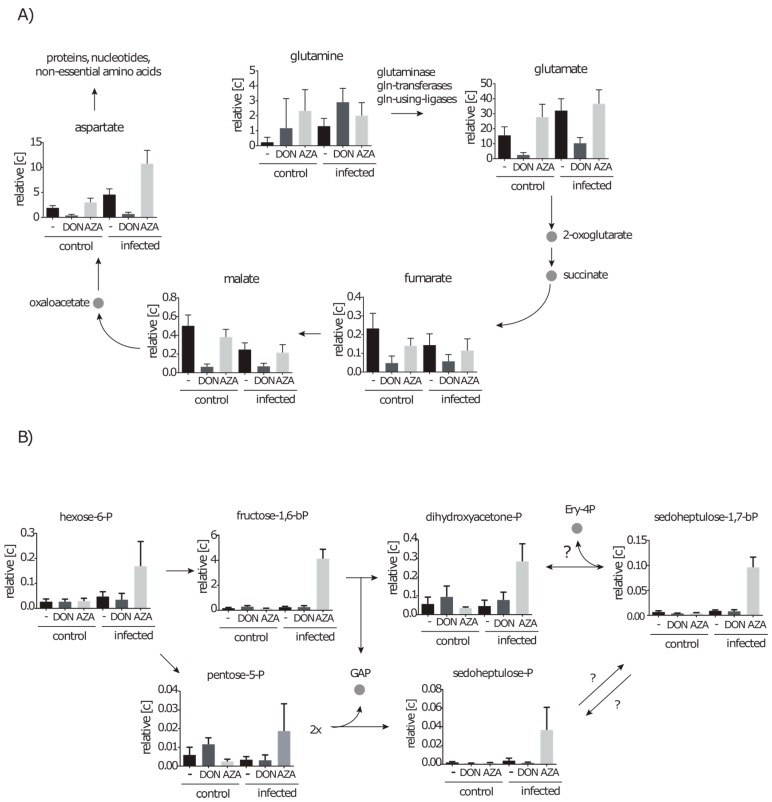
TCA-cycle intermediates are affected by DON and PPP intermediates are affected by azaserine during infection. Control cells and infected cells were treated with either DON or azaserine (AZA) for 12 h. Metabolites involved in: (**A**) glutaminolysis; and (**B**) carbon conversion pathways are displayed. Data are displayed in relative concentration as mean with standard deviation normalized on 10^7^ cells (*n* ≥ 7 out of at least four independent experiments).

Glutamate is generated during glutaminolysis, in glutamine transferase reactions, glutamine dependent ligase reactions or by the glutaminase reaction. Successive produced metabolites are fumarate and malate due to TCA-cycle reactions and aspartate by transamination of oxalacetate. In DON inhibited cells, the glutamate concentration dropped six fold and, consistently, malate, fumarate and aspartate were also decreased between five and eight fold (Figure 7A). Interestingly, in infected cells a similar three- to seven-fold drop in concentration was also observed for these metabolites. This suggests that some glutamate producing reactions are inhibited by DON under control conditions as well as under infection conditions. In addition, in infected cells treated with azaserine aspartate was increased more than two fold, suggesting that aspartate is still produced but less used in pathways such as purine and pyrimidine synthesis. Moreover, metabolites of upper glycolysis and the PP-pathway, in particular Frc-1,6-bP (16 fold), were strongly increased due to azaserine treatment during infection (Figure 7A,B). Other upstream and downstream metabolites namely hexose-6-P and dihydroxyacetone-P, pentose-P, sedoheptulose-P and sedoheptulose-1,7-bP were also found in elevated amounts between 4 and 10 fold.

### 2.5. De novo Purine and Pyrimidine Synthesis during Infection Is Strongly Reduced

By applying metabolic inhibitors, we were not able to reduce the intracellular nucleotide amounts under infection conditions. We therefore presume that either the inhibitor is not able to reach its target or the nucleotide synthesis is not active under infection conditions. To test if the purine and pyrimidine synthesis are active under infection conditions, we performed a labelling approach in which l-glutamine-amide-^15^N or d-glucose-^13^C_6_ was supplied to non-infected and infected cells. 

**Figure 8:**
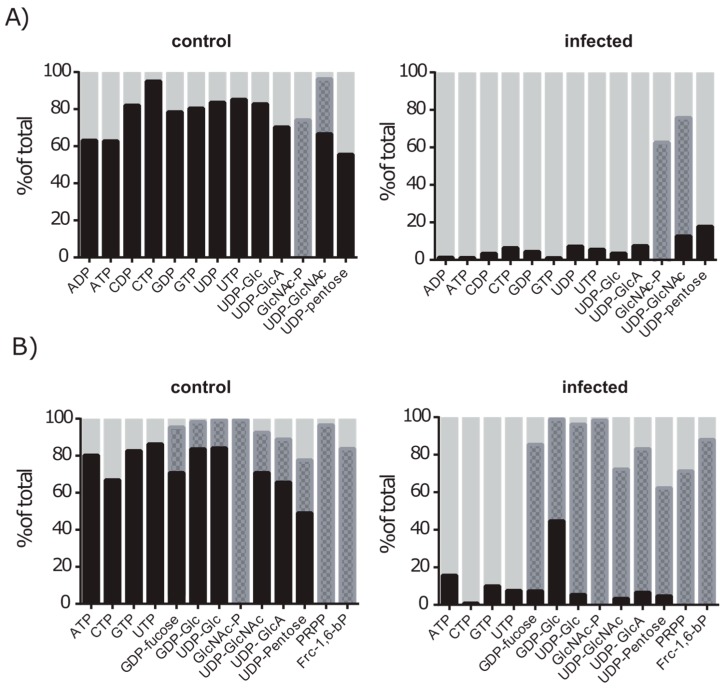
Incorporation of labelled precursor metabolites into purine and pyrimidine synthesis is strongly reduced during infection. Control cells and infected cells were cultured with either (**A**) l-glutamine-amide-^15^N or (**B**) d-glucose-^13^C_6_ for 12 h. Mean percentages of incorporation levels of labelled precursors are presented (*n* = 3). Proportions of complete labelled nucleotides and nucleotide-sugars are black, labelled sugar moieties are presented as grey pattern and unlabelled metabolite amounts are displayed as light grey.

The glutamine-amide group is transferred during the de novo synthesis of uridine once, adenosine and cytidine twice and three times in the case of guanosine. In addition, *N*-acetylation of glucose to GlcNAc does also require glutamine as a nitrogen donor. We analysed the metabolites with regard to the total labelling of these compounds and detected labelled nucleotides (ATP, ADP, UTP, UDP, GTP, GDP, CTP, CDP), GlcNAc-P and UDP-sugars (exact masses of analysed isotopes are provided in Supplementary Material Table S4) (Figure 8A). Between 60% and 95% of each nucleotide were found to be labelled after 12 h of cultivation. UDP-GlcNAc was detected in its complete labelled form and additionally with a labelled GlcNAc moiety only. However, in infected cells we could detect only minor amounts of the labelled nucleotides (1%–7%) at the same time of cultivation. Nevertheless, labelled isotopes of GlcNAc-P and UDP-GlcNAc with a labelled GlcNAc moiety were still found in similar or higher amounts compared to the control. This indicates that glutamine is still used for the formation of GlcN, but not for the nucleotide de novo synthesis. 

Since glucose delivers the carbon units for the ribose moiety of nucleotides via the PP-pathway, the production of nucleotides should also result in an absolute mass M + 5 in the labelling approach with d-glucose-^13^C_6_ (Supplementary Material Table S4). Indeed, the nucleotides ATP, UTP, GTP and CTP were dominantly present as the M + 5 labelled isotope (Figure 8B). Furthermore, glucose derived metabolites like Frc-1,6-bP, PRPP and GlcNAc-P were found in fully labelled state in high amounts up to 99%. In accordance with this, for metabolites with a glucose moiety such as UDP-Glc, GDP-Glc, and derivatives like UDP-GlcA and UDP-GlcNAc we identified the isotope M+6 (indicating a labelled sugar moiety) as well as the isotope M + 11 (indicating a labelled sugar moiety and ribose moiety). Twelve hours after infection, the nucleotide sugars were mainly found as M + 6 isotopes, whereas nucleotides were mainly detected in their unlabelled form. These data support the idea that nucleotide synthesis in infected A549 cells is switched off, while the formation of nucleotide sugars and pathways like glycolysis and the PP-pathway are still active.

Interestingly, growth inhibited A549 cells due to cytochalasin D treatment showed purine and pyrimidine labelling pattern in which CTP and UTP labelling decreased by 40% and 23%, respectively, but ATP and GTP only by 11% and 12% (Figure S1B). This indicates that growth arrest is only partly responsible for the reduced metabolic flux of the de novo nucleotide synthesis and other infection related processes are regulating this metabolic pathway. Moreover, glucose derived metabolites remained labelled as well as the sugar moiety of UDP-sugars.

In conclusion, these data indicate that purine and pyrimidine synthesis are active under control conditions in A549 cells as well as the further synthesis of nucleotide dependent metabolites such as nucleotide sugars. During the infection however, the nucleotide synthesis seems to be down regulated since incorporation in nucleotides of both d-glucose-^13^C_6_ and l-glutamine-amide-^15^N was strongly reduced during infection. The formation of UDP-sugars on the other hand, indicates that these reactions are not affected by the infection. Further ^15^N-labeling of GlcNAc-P and UDP-GlcNAc was also observed under infection conditions, confirming the results of the inhibitor assay, that GlcNAc formation is also active under infection conditions. 

## 3. Discussion

**Figure 9:**
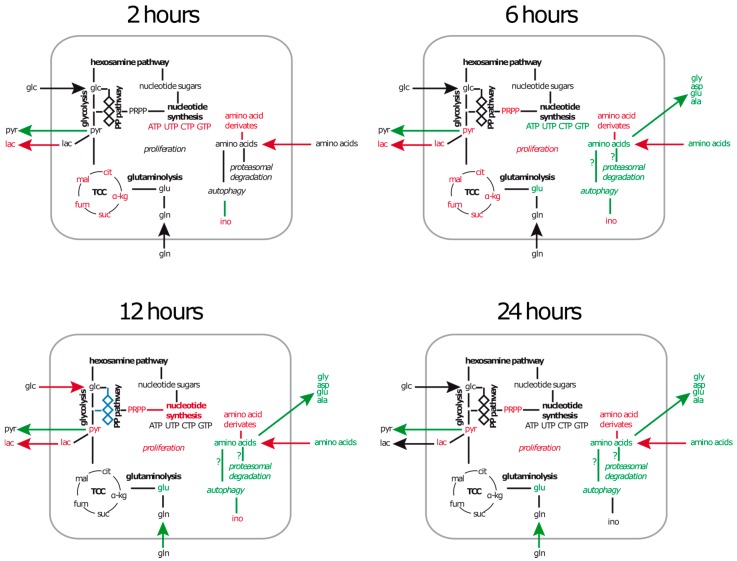
Key results of metabolic alterations and deduced pathways involved during infection. The summary graphic shows metabolic differences between control cells and cells after exposure to *S. aureus* measured at indicated time points. Names of metabolites are written in regular font, metabolic pathways in bold font and cellular processes in italic font. Green coloured labels indicate increased concentrations or higher activity and red labels indicate decreased concentrations or reduced activity. The pentose-phosphate pathway (PP-pathway) is coloured blue to indicate a changed flux of carbon during infection, which was only detectable when azaserine is present.

In this work, a metabolomics approach was used to investigate the metabolic consequences of *S. aureus* exposure in a human airway epithelial cell line. We observed time dependent changes in nutrient uptake, intracellular amino acid content and nucleotide synthesis (Figure 9). 

By monitoring growth and survival of A549 cells after exposure to *S. aureus*, we observed that the infection induces cell death in a certain proportion of the cells, but also restricts growth in the surviving population. Simultaneously, we observed a reduced uptake of essential nutrients from the cell culture medium. It is noteworthy, that cytochalasin D treated A549 cells also entered growth arrest but without a significant amount of cell death and the reduced uptake of amino acids. This strongly indicates, that growth arrest does not necessarily restrict nutrient uptake. Recently, it was shown that *S. aureus* infection inhibits proliferation of HeLa cells by a G2/M phase transition delay, most likely induced by the action of phenolsoluble modulin α (PSMα) [29,30]. α-type PSMs are peptides secreted by community-acquired MRSA such as USA300 that exhibit cytolytic activity and induce neutrophil activation [31]. A549 epithelial cells might also be affected by the action of secreted PSMα. When the intracellular bacterial load was strongly decreased, we observed after 48 h the resumption of proliferation and also the uptake of extracellular nutrients. This indicates that the cause for the growth inhibition is coupled to the amount of intracellular *S. aureus* cells. A lower amount of intracellular *S. aureus* would also result in a lower amount of secreted PSMα, which in turn would initiate growth again. Additionally, A549 cells were previously shown to enter growth arrest when infected with measles virus due to the upregulation of the Interferon Regulatory Factor 1 (IRF1) [32]. Moreover, lipoteichoic acids of *S. aureus* induce TLR2 signalling which leads to IRF1 upregulation [33] and therefore also might contribute to the growth arrest during infection. 

The early intracellular amino acid profile of infected cells showed an overall decrease in concentration. A similar general decrease in metabolite concentrations was recently observed by our group in airway epithelial cells treated with staphylococcal Hla [13]. Hla is a secreted virulence factor of *S. aureus*, which forms pores in the host cell membrane which has several effects on the host cell ranging from autophagy induction, alterations in cell signalling, cytoskeleton rearrangements and cytokine secretion [34,35,36]. It might also be responsible for the intracellular depletion of metabolites in this study, since USA300 is a potent producer of alpha toxin [37,38]. However, if Hla is responsible, it might also be secreted by *S. aureus* during the infection time, when bacteria and host cells were cultured together for 2 h. Other pathogens are also known to modulate cellular processes and metabolism from the extracellular space. In the case of pathogenic species from *Chlamydia, Xanthomonas*, *Pseudomonas*, *Ralstonia*, *Shigella*, *Salmonella*, *Escherichia* and *Yersinia*, this is accomplished by translocating effector molecules into the host cell cytosol via the type III secretion systems (reviewed by [39]). Thereby, many cellular processes that are directly or indirectly connected to the host cell metabolism are affected such as the proteasome, mitochondrial activity and intracellular signalling.

A common host reaction to intracellular pathogens is the upregulation of HIF-1 [40] which leads to enhanced glucose uptake and glycolytic activity. Indeed, evidence for an increased glucose metabolism via glycolysis by the infected host were found in various studies with *Mycobacterium tuberculosis* [41,42], *Listeria monocytogenes* [43] and *Chlamydia* spp. [44]. In our study, however, we observed a reduced glucose uptake and lactate secretion within the first 12 h of infection, which does not indicate a special role of HIF-1 activation or upregulation in A549 cells during *S. aureus* infection. We furthermore detected an increased amount of intracellular amino acids during the later time points after the infection in A549 cells. Since the uptake of amino acids was strongly reduced, enhanced nutrient consumption can be excluded. However, as an explanation cellular processes like autophagy or protein degradation seem appropriate. Proteasomal activity by the immunoproteasome upon infection stress can be induced by the cytokine IFN-γ [45]. Moreover, IFN-γ is released in response to *S. aureus* and immunoproteasomal mRNAs were found expressed in infected A549 cells [46]. Therefore the combination of an early alpha-toxin activity and a late host-response might lead to time dependent changes in the intracellular amino acid profile. From the perspective of the pathogen, this would result in a beneficial situation due to the elevated amount of free amino acids. Indeed, the chemical environment of the mammalian host cytosol supports growth rather of adapted bacteria [22]. Various intracellular pathogens have mechanisms to elevate the intracellular amino acid content to their benefit, indicating that especially intracellular amino acids are not concentrated enough for bacterial replication. *Francisella tularensis* induces macroautophagy in the host and *Legionella pneumophila* promotes proteasomal degradation to elevate the intracellular amino acid amount [47,48]. It is not clear, whether *S. aureus* induces one of the above-mentioned processes for the same reason, but *S. aureus* is known to induce autophagy in host cells [19,35,49]. In line with these findings we detected strongly reduced amounts of intracellular *myo*-inositol, which was demonstrated to induce autophagy in an mTOR-independent manner [50]. However the second metabolic marker for this alternative autophagy induction namely inositol-1,4,5-trisphosphate was detected only in trace amounts under both conditions at all times. The stringent response is a bacterial indicator for a non-sufficient amino acid supply in the current environment, by which the alarmone (p)ppGpp is synthesized and regulates more than 150 genes in *S. aureus* [51]. Three (p)ppGpp synthetases RSH, RelP and RelQ are present in *S. aureus*. RSH contains an amino acid sensing domain, whereas the latter two enzymes lack this domain and are most likely involved in the response to cell wall stress [52]. Recently, it was shown, that RSH is downregulated in intracellular *S. aureus* cells but the other two (p)ppGpp synthetases are upregulated [46]. This is in line with our finding that the amount of amino acids is increasing in the host cytosol, whereas cell wall stress due to phagosomal activity is likely to occur.

Our study revealed that, beside amino acids, the amounts of the nucleotides GTP and CTP are temporarily elevated in cells exposed to *S. aureus*. Recently, the CTP-synthetase of T-cells was found to play an important role in proliferation after T-cell activation [53]. Moreover, in murine lung cells infected with *Mycobacterium tuberculosis* nucleotide concentrations were elevated and also connected to active replication of the host cells [41]. To test the activity of nucleotide biosynthesis pathways, we used azaserine and DON as metabolic inhibitors. We identified their potential target sites due to the amounts of educts and products of glutamine utilizing reactions. Our data suggest that especially reactions in purine and pyrimidine synthesis were inhibited by DON and azaserine. Similar metabolic patterns were previously described in mouse leukaemia cells for the CTP-synthetase, PPAT and PFAS [27]. For other target sites we observed contrary results. Azaserine and DON are frequently described as potent inhibitors of the hexosamine biosynthesis pathway by inhibiting GFAT [27,54,55,56]. Our study confirmed the inhibitory action in this pathway for DON, whereas treatment with azaserine showed only minor effects. We additionally identified the GMP-synthetase as a potential target site of DON by detecting the educt XMP in elevated concentrations when the inhibitor was present. The inhibitory action of DON under control conditions results in depletion of CTP, GlcNAc and their derivatives, which is likely to lead to growth inhibition. The cause of azaserine induced growth inhibition however is not as clear. Although the FGAR-synthetase was found to be inhibited, purine and pyrimidine amounts were not different compared to control cells. However, we detected a so far unknown metabolite (Supplementary Material Table S3) that was strongly increased due to azaserine treatment, but further studies are needed to identify this metabolite and resolve its role in cell proliferation. Post infection, however, the inhibitory effect was only marginally visible. Moreover, the CTP amount was still elevated in inhibited infected cells compared to control cells. By using a labelling approach we were able to demonstrate that in A549 cells: (i) the carbon flux into the nucleotide synthesizing pathways is strongly reduced; and (ii) glutamine utilizing enzymes in purine and pyrimidine pathways including the CTP-synthetase show significantly lower activity. This may also explain the secretion of glycine and aspartate by infected cells. Glycine production contributes to the THF-C_1_-pool and is especially needed for synthesis of purines. Moreover, aspartate is an important precursor for both purine and pyrimidine synthesis. If utilization of these amino acids is strongly reduced, the excess amounts might rather be secreted. Our data therefore strongly indicate that the growth arrest induced by *S. aureus* is not due to an intracellular nutrient limitation, and that nucleotide de novo synthesis and utilization of nucleotides is strongly reduced in cells after being exposed to *S. aureus*. Moreover, we could demonstrate that a reduced growth rate of A549 cells is not sufficient to explain the drastically lower flux of carbon into de novo nucleotide synthesis and other infection related regulations are most likely responsible. However, this does not apply to the hexosamine biosynthesis pathway. It remained clearly inhibited in infected cells treated with DON and moreover was found to be active in our labelling approach. UDP-GlcNAc is the end product of the HBP and the substrate for the *O*-GlcNAc transferase, which transfers the GlcNAc moiety to serines or threonines of target proteins, thereby facilitating the post-translational modification of *O*-GlcNAcylation [57]. The HBP relies on glucose and glutamine to build up UDP-GlcNAc and is therefore a proposed sensor of the nutrient situation [58]. In cancer cells, such as those used in this study, uptake of glucose and glutamine is strongly elevated leading to an increased flux into the HBP and driving *O*-GlcNAcylation [58,59]. Exposure of A549 cancer cells to *S. aureus* did not change the nutrient availability and also glycolysis and glutamine uptake were still active. The increased glutamine uptake and the constant uptake of glucose and secretion of alanine, pyruvate and lactate support this idea. We therefore suggest that the HBP is not affected by the *S. aureus* infection or the resulting host response, but rather by the presence of its precursors.

As a surprising result we found, that only infected cells exposed to azaserine show strongly increased amounts in metabolites involved in central carbon metabolism especially upper glycolysis and pentose-phosphate pathway. This indicates that the carbon flux is somehow changed due to the infection but only visible when azaserine is present. Furthermore, it suggests that azaserine affects a pathway in which the carbon is channelled when cells are infected, either directly or indirectly. 

In summary, our metabolomics approach provides for the first time information about the dynamics in metabolism of A549 cells after being exposed to *S. aureus*. We could confirm that infected cells enter growth arrest and moreover reduce nutrient uptake. We furthermore identified intracellular metabolic pattern, which represent the host metabolome but also the metabolic environment for intracellular bacteria such as *S. aureus.* Moreover, we present clear evidence for a shutdown in nucleotide biosynthesis in A549 cells after exposure to *S. aureus*. How this shutdown is regulated is still unclear, but it pinpoints the close interplay of the infection by a pathogenic bacterium and the host cell metabolism.

## 4. Materials and Methods 

### 4.1. Cell Culture and Infection

For analysis of the cell culture medium 60 mm cell culture dishes were used. A549 cells were purchased from the American Type Culture Collection (ATCC-107, Leibniz Institute DSMZ-German Collection of Microorganisms and Cell Cultures, Heidelberg, Germany).

For extracellular metabolome samples 0.9 × 10^6^ cells were seeded in 4 mL RPMI 1640 R7509 medium (obtained from Sigma Aldrich, Munich, Germany) (2 mmol/L glutamine and 10% FCS were added, both obtained from Sigma Aldrich) 24 h before infection. Control cells were cultivated for 24 h until maximum confluence was reached and infected cells were cultivated over a time period of 72 h with a medium change every 24 h to prevent nutrient limitation. 

*Staphylococcus aureus* USA300 LAC (obtained from the strain collection of the Institute for Microbiology, Ernst-Moritz-Arndt University Greifswald, Greifswald, Germany) was cultivated in RPMI medium supplemented with 2 mmol/L glutamine and trace elements as described for other *S. aureus* strains [60]. On the day before infection a pre-culture was inoculated from a glycerine-stock culture. The main culture was inoculated from the pre-culture with an optical density of 0.05 at 560 nm and was cultivated to an optical density of 0.5. *S. aureus* cells were washed with PBS and resuspended in RPMI supplemented with 1% FCS to an optical density of 0.5. For infection, A549 cells were washed and 1.5 mL infection medium (2 mmol/L glutamine and 1% FCS) was added containing 450 μL of a *S. aureus* cell suspension with an optical density of 0.5 corresponding to a multiplicity of infection of approximately 50:1. After 2 h under standard growth conditions (37 °C and 5% CO_2_) the cell monolayer was washed twice with PBS and 2.25 mL fresh RPMI medium supplemented with 2 mmol/L glutamine, 10% FCS, lysostaphin (20 μg/mL, obtained from Sigma Aldrich) and gentamicin (100 μg/mL) were added (incubation medium). Cells were incubated under standard conditions for 2 h to 72 h with a medium change every 24 h. For intracellular metabolome samples the infection was performed as described above, but in 150 mm cell culture dishes. For that matter 6.0 × 10^6^ cells were seeded in 25 mL RPMI medium 24 h before infection. For infection the medium was changed with 10 mL infection medium containing 3 mL *S. aureus* suspension with an optical density of 0.5. After infection time, cells were washed and 15 mL of incubation-medium were added. 

To determine the cell number and viability of cells and the number of intracellular *S. aureus* cells, cells where washed and 2 mL of accutase (A549 cells) (obtained from Sigma Aldrich) were added for 5 min to detach the cells. To elucidate the cell number, an aliquot of the cell suspension obtained was mixed with trypan blue and counted using a Countess Automated Cell Counter (Invitrogen, Carlsbad, CA, USA). An aliquot of the infected A549 cell suspension was diluted in several steps and further plated on LB-Agar plates to determine the colony forming units of living *S. aureus* cells. 

### 4.2. Metabolic Inhibitors and Labelled Precursors

To inhibit glutamine dependent metabolic reactions, we applied two glutamine analogues namely 6-diazo-5-oxo-l-norleucine (DON) with a final concentration of 0.5 mmol/L and azaserine (both obtained from Sigma Aldrich) with a final concentration of 0.028 mmol/L to the incubation medium for 12 h. In our ^15^N labelling approach we used RPMI 1640 R7509 medium as incubation medium as described above but replaced unlabelled glutamine with 2 mmol/L of l-glutamine-(amide-^15^N) (obtained from Sigma Aldrich). For ^13^C labelling we used RPMI 1640 R1383 medium as incubation medium with 10% FCS, 2.0 g/L sodium bicarbonate and 11.0 mmol/L d-glucose-^13^C_6_ (obtained from Sigma Aldrich). For growth inhibition experiments cytochalasin D (Sigma Aldrich) was used in a final concentration of 1 μg/mL for 12 h.

### 4.3. Extracellular and Intracellular Metabolome Samples

Extracellular metabolome samples were generated by collecting the cell culture medium at 2 h, 4 h, 8 h, 12 h and 24 h of control and additionally at 48 h and 72 h of infected cells. For that matter 2 mL of the supernatant from the culture were sterile filtered and directly frozen at −20 °C. 

At time points 2 h, 6 h, 12 h, and 24 h cells were harvested according to a protocol described previously with a minor modulation [13]. In brief, cells were washed 4 times with ice cold NaCl solution (130 mmol/L), then the internal standard for GC-MS analysis ribitol (40 nmol) was added to the plate before 10 mL of ice-cold methanol were added. Cells were scraped, transferred into a 50 mL tube and placed on ice. The cell culture dish was washed with 10 mL ice-cold double distilled water, which was added to the 50 mL tube and subsequently shock frozen in liquid nitrogen and stored until extraction at −80 °C. Extraction of metabolites was carried out as described [13]. In brief, samples were thawed on ice and the internal standard for HPLC-MS measurements camphorsulfonic acid (5 nmol, obtained from Sigma Aldrich) was added. Next 2 mL of chloroform were added and the sample was mixed and placed on ice for 10 min. The sample was centrifuged for 10 min at 3000× *g* at 4 °C and the aqueous phase was collected. Ten millilitres of ice cold water were added to the organic phase and the sample was again mixed and centrifuged for 10 min at 3000× *g* at 4 °C. The aqueous phase was collected and united with the previous aqueous extract. After extraction the samples were split 1:1 for GC-MS and LC-MS analysis, frozen and lyophilized. 

### 4.4. ^1^H-NMR Spectroscopic Analysis and Data Analysis

Cell culture medium samples were thaw at room temperature. Four hundred microlitres of the sample were mixed with 200 μL of a sodium hydrogen phosphate buffer (0.2 mol/L, pH 7.0) including 1 mmol/L trimethylsilyl propanoic acid made up with 50% D_2_O for ^1^H-NMR analysis as described [61]. A Bruker AVANCE-II 600 NMR spectrometer operated by TOPSPIN 3.2 software was used (both Bruker Biospin, Rheinstetten, Germany,). For qualitative and quantitative data analysis AMIX^®^ (Bruker Biospin, version 3.9.14) was used. The AMIX Underground Removal Tool was used to correct the baseline of obtained NMR-spectra. Thereby the following parameters were applied: left border region 20 ppm and right border region −20 ppm and a filter width of 10 Hz. The region of noise that was used for final baseline correction was between 5.5 ppm and 5.6 ppm. Absolute quantification was performed as described [61].

### 4.5. LC-MS Setup and Analysis

For LC-MS analysis a time of flight (micrOTOF) mass spectrometer (Bruker Daltonik, Bremen, Germany) was used with a setup as described [62]. In brief, we used a SymmetryShield RP18 column (Waters) and used an aqueous mobile phase with tributylamine as ion-pairing reagent and methanol as a second mobile phase. In doing so, we were able to analyse mainly negatively charged compounds such as nucleotides, sugar phosphates and nucleotide sugars. Identification of peaks was carried out by comparison of *m*/*z* values and retention time of signals with an in-house database, the human metabolome database (HMDB) and the Metlin database. Metabolites that could not be verified via measurements of reference compounds due to commercial availability were identified by the accurate mass only if the deviation was <5 ppm (Supplementary Material Table S5). Metabolite quantification was done by QuantAnalysis^®^ (Bruker Daltonik). Peak areas of extracted ions were normalized to the internal standard area of camphorsulfonic acid and further normalized to the respective cell number.

### 4.6. GC-MS Setup and Analysis

Lyophilized samples were derivatised as described [63], using a two-step derivatization method with MeOX (Sigma-Aldrich) and MSTFA (Chromatographie Service GmbH, Langerwehe, Germany). Derivatisation method, type of column and oven program were optimized to analyse mainly small polar compounds such as amino acids, carbohydrates and organic acids. For identification and quantification of metabolites a GC-MS method was used as described [62]. Qualitative and quantitative analysis were performed using ChromaTOF software (LECO Corporation, St. Joseph, MI, USA). Identification of peaks was carried out by comparison of mass spectra and retention time of signals with an in-house database and the NIST database. The relative metabolite concentrations were obtained by relating the signal area of each metabolite to the internal standard ribitol and further to the respective cell number.

### 4.7. Statistics and Data Visualisation

Graphs were generated and tests of statistical significance were performed using Prism (version 6.01; GraphPad Software, La Jolla, CA, USA). Statistical significance was calculated by multiple unpaired *t*-tests of at least 3 biological replicates. *p*-values ≤ 0.01 were considered as being statistically significant. 

Extracellular data are presented as relative concentration difference Δ[c] between the indicated time point t_x_ and t_0_, normalized to changes in cell number (cell number t_x_/cell number t_0_). Values are presented as bar charts or colour coded charts, which were created using MeV v4.8.1 [64] with the following settings for hierarchical clustering: optimized gene leaf order, euclidean distance metric and average linkage method. 

Intracellular data are presented as volcano plots and word clouds [65], colour coded charts or bar charts. For relative concentrations, computed values of the signal area were normalized on the corresponding internal standard and the cell number. For calculation of fold changes, missing values were replaced with half the value of the lowest abundant metabolite that was analysed (Supplementary Material) and assumed to be the detection limit.

## Supplementary Materials

The following are available online at http://www.mdpi.com/2218-1989/6/4/41/s1, Figure S1. (A) Uptake and secretion of amino acids by A549 cells after 12 h of cytochalasin D treatment. Extracellular concentrations changes of amino acids were normalized on the proliferation rate ([c_tx_-c_t0_])/(cell number_tx_/cell number_t0_). Data are presented as mean (*n* = 5) in a colour coded chart. Red fields indicate an increase in the extracellular concentration and blue fields indicate a decrease of the metabolite concentration compared to the initial concentration. (B) Mean percentages of incorporation levels of D-glucose-^13^C_6_ of A549 cells without and with 12 h treatment with cytochalasin D are presented (*n* = 4). Proportions of complete labelled nucleotides and nucleotide-sugars are black, labelled sugar moieties are presented as grey pattern and unlabelled metabolite amounts are displayed as light grey; Table S1: extracellular data, Table S2: intracellular data (infection); Table S3: intracellular data (metabolic inhibitor assay); Table S4: intracellular data (metabolic labelling assay); Table S5: molecular masses of identified metabolites
