# Supplementary material for: Staphylococcus aureus Infection Reduces Nutrition Uptake and Nucleotide Biosynthesis in a Human Airway Epithelial Cell Line
Source: Metabolites. 2016 Nov 9;6(4):41. doi: 10.3390/metabo6040041 (PMC5192447; doi:10.3390/metabo6040041)
Supplement: Supplementary file 1 [file metabolites-06-00041-s001.zip › metabolites-157069-for publishing-supplementary/metabolites-157069-for publishing-supplementary-Figure.docx]

Supplementary Materials: *Staphylococcus aureus* Infection Reduces Nutrition Uptake and Nucleotide Biosynthesis in a Human Airway Epithelial Cell Line

Philipp Gierok, Manuela Harms, Karen Methling, Falko Hochgräfe and Michael Lalk


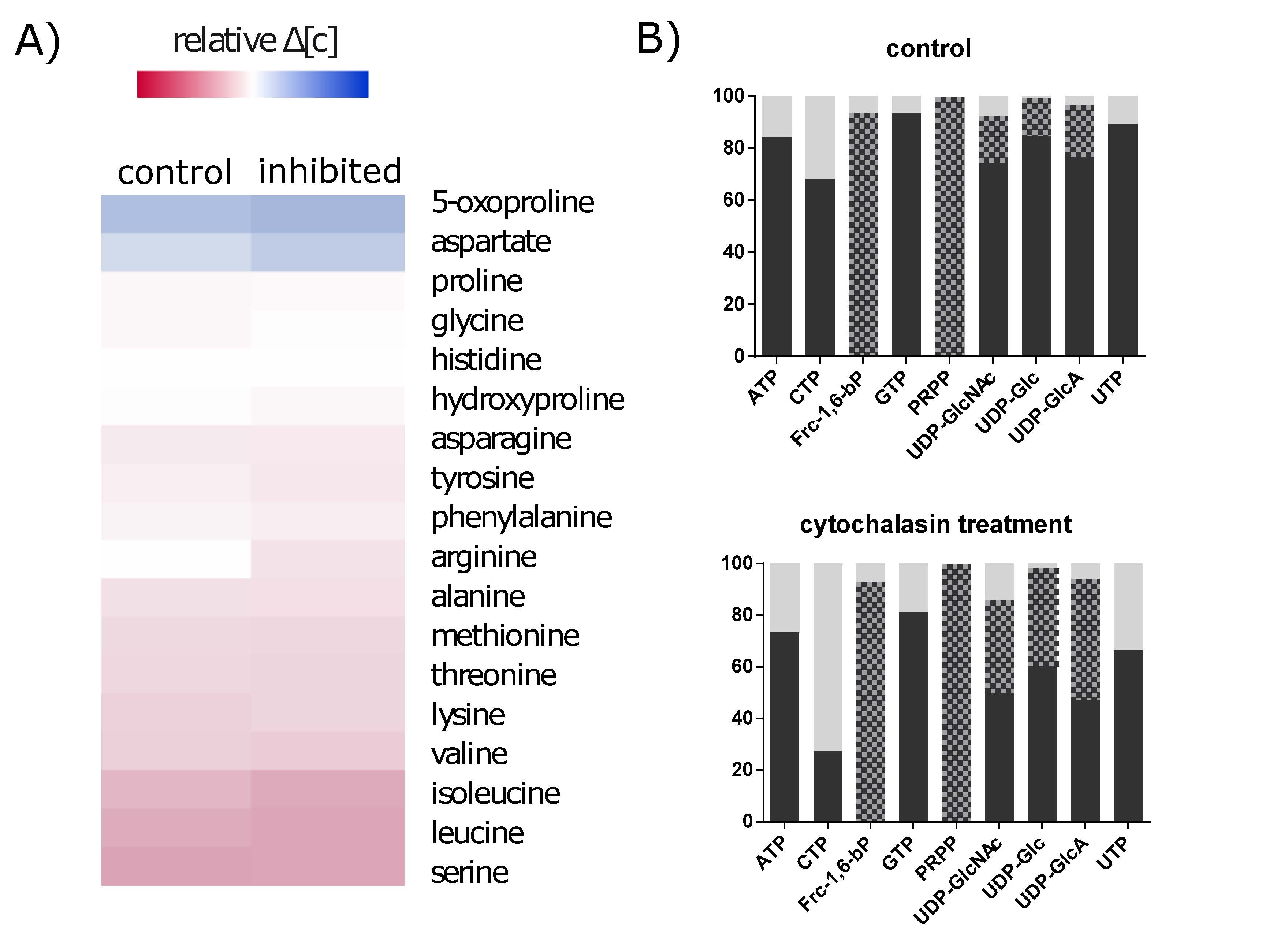


**Figure S1.** (**A**) Uptake and secretion of amino acids by A549 cells after 12 h of cytochalasin D treatment. Extracellular concentrations changes of amino acids were normalized on the proliferation rate ([c_tx_-c_t0_])/(cell number_tx_/cell number_t0_). Data are presented as mean (*n* = 5) in a colour coded chart. Red fields indicate an increase in the extracellular concentration and blue fields indicate a decrease of the metabolite concentration compared to the initial concentration. (**B**) Mean percentages of incorporation levels of D-glucose-^13^C_6_ of A549 cells without and with 12 h treatment with cytochalasin D are presented (*n* = 4). Proportions of complete labelled nucleotides and nucleotide-sugars are black, labelled sugar moieties are presented as grey pattern and unlabelled metabolite amounts are displayed as light grey.
